# Study of Microwave-Active Composite Materials to Improve the Polyethylene Rotomolding Process

**DOI:** 10.3390/polym15051061

**Published:** 2023-02-21

**Authors:** Giorgio Luciano, Maurizio Vignolo, Elisabetta Brunengo, Roberto Utzeri, Paola Stagnaro

**Affiliations:** Istituto di Scienze e Tecnologie Chimiche “Giulio Natta”—SCITEC, National Research Council of Italy, Via de Marini 6, 16149 Genova, Italy

**Keywords:** rotomolding, microwave heating, microwave-active materials, polyethylene processing

## Abstract

The present paper reports on the formulation and characterization of composite coating materials susceptible to microwave (MW) heating to investigate their application in making the rotomolding process (RM) more energy efficient. SiC, Fe_2_SiO_4_, Fe_2_O_3_, TiO_2_ and BaTiO_3_ and a methyl phenyl silicone resin (MPS) were employed for their formulations. Experimental results showed that the coatings with a ratio of 2:1 *w*/*w* of inorganic/MPS are the most MW-susceptible materials. To test the coatings in working mimicking conditions, they were applied to molds, and polyethylene samples were manufactured by MW-assisted laboratory uni-axial RM and then characterized by calorimetry, infrared spectroscopy and tensile tests. The results obtained suggest that the coatings developed can be successfully applied to convert molds employed for classical RM process to MW-assisted RM processes.

## 1. Introduction

World is implementing sustainable manufacturing and the consequence of effective utilization of resources is increasing productivity. Among the several ways of improving resource effectiveness is avoiding use of resources in the first place and reducing its footprint is paramount [1]. Plastic industry is an energy-intensive one requiring a high volume of electric power for thermal energy where energy use accounts for 5–10% of total production cost [2].

Among the plastic processing methods, rotational molding, also known as rotomolding (RM), is a casting and molding technique useful to produce hollow plastic items of medium to large size [3,4,5] and involves manufacturing in food and agriculture sector, industrial application, automotive, containers, consumer items and toys. RM is a relatively small part of the plastics industry practiced by approximately 2500 companies around the world. It consumes approximately 0.7% of the total volume of the world production of plastics [6,7,8,9] and references therein. To enhance the energy performance of the RM process, the setup required for heating the molds represents a key factor.

In the present paper we focused on enhancing the performance of the process by heating via microwave (MW) irradiation in alternative to using gas or electric ovens. For this aim, we formulated composite materials based on MW-susceptible inorganic compounds (MWSIC) and a methyl phenyl silicone resin to modify conventional RM molds.

In MW heating, also known as dielectric heating, we have a direct transfer of energy (which travels at the speed of light). Consequently, a large amount of power can be saved as well as process times and operational space, considering the physical phenomena involved in the thermal conduction, which is inversely proportional to the square of the distance in the between of the energy source and material to be mold [10].

MW heating is obtained by irradiating active materials in the microwave energy range (10^−3^ kJ/mol), which is too low of a value for chemical bonds cleavage, but it is sufficient to heat or melt the material by conversion of electromagnetic energy of MW into thermal energy (vibrational motions of chemical bonds). For this reason, the involved materials need to have permanent dipoles (dielectric material) to work well.

In this study, attention was focused on the investigation of MW-active coating materials to be adopted to make the molds suitable for MW heating and thus make it possible to manufacture objects in polyethylene (PE), which is not a dielectrically active material.

Several formulations based on MWSIC were tested in terms of their response to MW irradiation. Measurements of electric power absorbed by selected composite formulations during the imposed microwave cycle were performed and compared to the power needed for corresponding resistive irradiation. PE samples were prepared by MW-assisted uni-axial rotomolding in a lab oven and their properties compared to those of commercial or differently prepared samples.

## 2. Materials and Methods

### 2.1. Materials

Five different MWSIC materials in powder form, namely, silicon carbide (SiC), iron (II) silicate (Fe_2_SiO_4_), iron (III) oxide (Fe_2_O_3_), titanium (IV) oxide (TiO_2_), barium titanate (IV) (BaTiO_3_), were tested. Furthermore, it was studied also the effect of different grain size: 35 μm (Fine) and 70 μm (Coarse) for SiC (SCF and SCC, respectively) and 35 μm (Fine) and 500 μm (Coarse) for Fe_2_SiO_4_ (ISF and ISC) (see Table 1).

Except for ISF and ISC kindly supplied by Slide S.r.l. (Italy), all other inorganic materials were purchased from Sigma-Aldrich (now Merck). Grain size of each substance was obtained from the corresponding label on the commercial container.

To select the most effective materials, that are most capable of efficiently absorbing the MW irradiation heat transfer measures were performed on pelletized samples (discs of 1.2 mm diameter and 0.5 mm thickness) prepared by mixing each MWSIC powder typology with a high-temperature-resistant methyl phenyl silicone resin (MPS). The resin used for the pellet formulation was a commercial two-component silicone elastomer resin (BLUESIL ESA 7252 A&B Italy), fast curable at r.t. and endowed with outstanding flame resistance and good thermal conductivity. The MWSIC powder:MPS resin ratio used was 2:1 (*w*/*w*).

To gather more information about the materials which can be used as molds, different cylindrical containers were coated with the MW-active composite materials. Aluminum (AL, 50 g), stainless steel (SS, 70 g) and glass (GL, 170 g) containers were tested.

A commercial PE grade (Plastene R210, Poliplast S.p.A. Italy, kindly supplied by Slide S.r.l.) in form of powder (mean dimension 410 µm), was employed for the tests in lab. Plastene R210 has melt flow index (ISO 1133, 190 °C, 2.46 kg) 6.25 g/10 min and density 0.936 g/cm^3^ (ISO 1183).

For de-molding of PE objects TECNOSIL 21 (SOL TECNO S.r.l., Italy), a technical silicone oil employed in industrial production, was used as detaching agent.

For comparison purposes, two other polyethylene types, namely, Riblene and Kartell jar, were tested by mechanical tensile tests, DSC and ATR-FTIR.

### 2.2. Methods

For the MW-assisted RM process we used a mold coated with MW-susceptible material while in the conventional RM process the mold was uncoated.

A MW oven SAMSUNG M/O 20LT GE71A of 20 L in volume as internal space, operating at 2.45 GHz frequency and at 750 W in power consumption, also equipped with a grill (1100 W in power consumption), was used in heating cycles both with the microwave and the resistance irradiation, for comparison purpose.

To have more information about electric energy saving, the same process parameters (rpm, time, PE powder amount and mold) were adopted to simulate a PE molding using MW irradiation or the resistance of the oven (grill). In each process the absorbed electric current (I) was measured, as well as the voltage value (V) by a digital multimeter (Powermeter GBC KDM-360CTF), respectively, connected to the circuit in series as a galvanometer or in parallel mode as a voltmeter.

The heat transfer measurements were performed positioning each composite pellet on the center of a Teflon plate fixed to the motor axis of the MW oven; the heating cycle was tested at 750 W power both for 5 and 1.5 min.

After the MW irradiation, each pellet was immediately quenched into 20 g of demineralized water at room temperature contained in a plastic Petri with a stirring bar under movement. A Hg thermometer (±0.5 °C in sensibility) was adopted for the temperature measurements.

The composite pellets were rapidly transferred from MW oven to water using high-temperature-resistant and low-thermal-conductivity plastic tweezers.

Microwave-active ISC powder was then chosen as the most suitable to prepare the coatings to be adhered to the molds using the same weight ratio (2:1 powder:resin) previously used for the testing pellets (in this case, 60 g ISC:30 g MPS).

The molding process of PE was tested by performing MW heating cycle of 300 W for 13 min, with a uni-axial rotational movement on the mold axis and a speed of 2 rpm. This speed, as well as the rotation of the mold even during the cooling phase, was possible by replacing the original oven motor (6 rpm). Such a low rotational speed was useful to have sufficient contact time between PE powder and the hot mold internal surface and thus improve the heat exchange.

For each test, 15 g of PE powder were utilized.

Uni-axial tensile measurements on produced PE objects and reference PE counterparts were performed with a Shimadzu ASG-X 10 kN universal machine operating at r.t. on 5 dog-bone specimens (for each PE type) prepared in shape and dimension as requested by ISO 527 (1–5) using a dog-bone shaped mold or a die cut from a previously die-cast PE plate.

To obtain the dog-bone test specimens from uni-axial RM process assisted by MW, a cylinder of PE was prepared from Plastene powder by applying a MW cycle of 10 min at a power energy of 750 W, and a cooling time of about 30 min at a speed of 6–8 °C/min was adopted.

As a first term of comparison, a PE plate was prepared from the same Plastene powder using a Colling press (Laboratory Platen Type P200 bar) and applying the following thermal cycle: heating up to 300 °C, heating rate 10 °C/min, 10 min isothermal at 100 bar, cooling rate at 10 °C/min to room temperature. As a second term of comparison, a commercial PE item, namely, a Kartell PE jar (1000 mL), was employed.

FTIR spectra of PE samples (commercial and rotomolded) were recorded using a Perkin Elmer FTIR Spectrum Two™ spectrophotometer. FTIR spectra were acquired in attenuated total reflection (ATR) mode in the range of 4000–400 cm^−1^.

Calorimetric analysis was performed with a DSC Mettler 821^e^ instrument on specimens of 10 mg (cut from the manufactured samples) applying a heating–cooling–heating cycle in the range 100–240 °C under N_2_ and at a scan rate of 10 °C/min (EN ISO 11357-1-3).

The morphology and elemental analysis of different MW-active coating formulations were performed by scanning electron microscope (SEM) equipped with a probe for energy-dispersive X-ray analysis (EDX); in detail, it is a HITACHI TM3000 benchtop SEM (15 kV).

## 3. Results and Discussion

The main objective of the present study is to prove the easiness of the conversion of the molds employed in a standard oven to the new coated ones for the MW-assisted RM process. For this purpose, we tested (by heat transfer measurements) the ISC/resin (2:1 ratio) composites on molds made of different materials.

Silicone elastomer resin was chosen due to the working temperature conditions ranging from ambient temperature to 400 °C. It is well established that silicon resins over a wide range of temperatures and they also acknowledged for their fire resistance properties. Moreover, the elastomeric nature of MPS leads to good adherence of the coatings to the molds.

The MWSIC materials of this study were chosen for their dielectric constant (relative permittivity *ε_R_*) and dissipation factor values acquired from the literature and reported in Table 2 for room temperature [8,9,10,11,12,13,14,15,16,17,18,19,20,21] and considering negligible the change in value for frequencies higher than 100 MHz [15].

Dielectric constant *k*′ (and permittivity) and dissipation (loss) factor are related by Equation (1).

k* = *k*′ − j*k*″ (1)

In addition,

*k′* = ε*′/*ε_0_ (2)

and

*k″* = ε*″/*ε_0_ (3)

where the terms *ε*′ and *ε*″ represent, respectively, the real and imaginary part of complex permittivity ε* and ε_0_ is the vacuum permittivity.

Because *k*′ can be related to the material capability to storage electrical energy and *k*″ represents its capability to dissipate electrical energy, we can define the ability of a material to convert the microwave radiation into heat as the tangential loss ratio:

tan δ = ε″/ε′(4)

Reference materials used in the experiment and the relative parameters for water, PVC, glass, and PTFE are reported in Table 2.

Because the literature data referred to different measurement parameters, such as temperature, frequency, shape, size and so on, heat transfer measurements were here performed in order to establish the response of the chosen compounds in the laboratory environment.

The values of heat exchanged *Q* of compounds pelletized with the silicone resin were calculated using Equation (5) and are reported in Table 3.

The amount of heat (*Q*) was estimated by Equation (5)

Q = m·c·(ΔT) [J](5)

where ΔT is the difference between the temperature value of the water before and after immersion of the pellet, m correspond to the mass of water and c is its specific heat value (4.18 J·g^−1^·K^−1^). Irradiation time was checked using a precision chronometer.

The results indicate the higher response (expressed as exchanged heat between the material and water used as reference) was obtained by ISC/F samples, followed by SCC/F and IO. The role of the graininess in the response was evaluated both for iron silicate and silicon carbide. In the first case, a difference of 30% in the particles dimension caused a 30% difference in exchanged heat. For the silicon carbide, no effect was found due to the graininess (fine or grain silicon carbide gave identical results).

Among the main objectives of the project one of the most important was the easiness of the conversion of the molds employed in a standard oven to the new coated ones for the MW-assisted RM process. For this purpose, we tested (by heat transfer measurements) the ISC/MPS (2:1 ratio) composites on different materials used for the molds, namely, aluminum, stainless steel and glass (here labeled AL, SS and GL, respectively).

Absorbed power (P_abs_) was calculated in W units with Equation (6), where V is the voltage and I the electric current, converted into W per hour (Wh) to have a direct comparison, considering the impulse time for MW irradiation and the continuous power adsorption for the resistance irradiation.

P_abs_ = V × I [W] (6)

In Table 4 and Table 5, the resultant absorbed power values are reported.

All three molds, coated with different materials (AL, SS and GL), reached a maximum temperature of 160 °C under MW and 125 °C when employed under a resistance regime.

**Figure 1:**
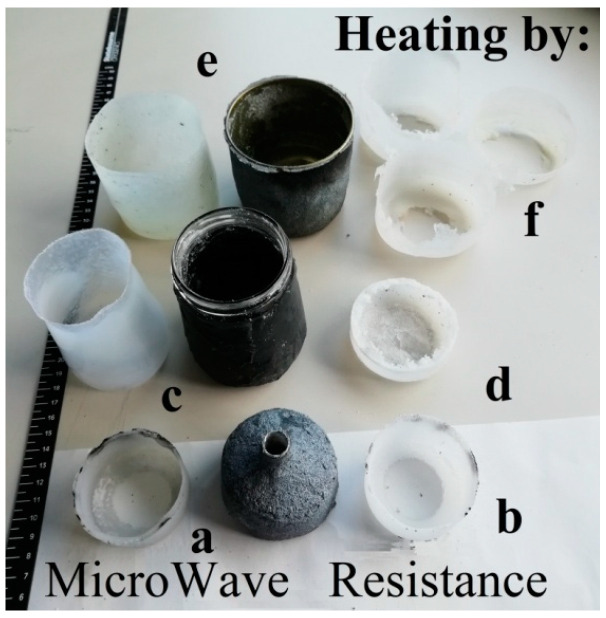
The PE products of molding tests and respective molds (in the between): a, c and e from the MW heating of coated AL, GL and SS molds, respectively; b, d, and f from the resistance heating the corresponding molds.

The results highlighted that by employing a MW regime, there is the possibility of melting PE and reaching the target temperature for effective RM laboratory processing (as shown in Figure 1).

Analogous results were obtained for the other materials employed. In all cases, MW-assisted RM ensured a more homogeneous heating of the mold with consequent formation of objects, while the heterogeneous heating obtained by resistance irradiation led to RM-manufactured objects severely failed (missing parts, high surface roughness and residual non-melted raw powder material).

In Table 6, the weights of each PE product in the respective irradiation regime are reported to sustain previous affirmations.

**Figure 2:**
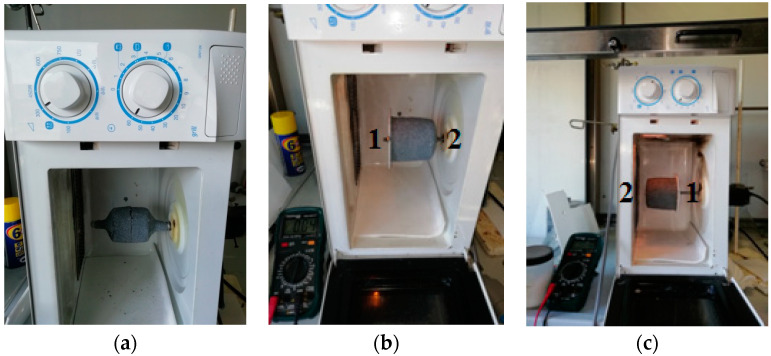
Different materials and configurations of molds: (**a**) AL, (**b**) GL and (**c**) SS in different configurations: white PTFE cap (labeled as 1) on the left side or on the right, respectively. The side of the MW-active material is labeled as 2.

Reported in Figure 2 are the setups used to study the influence of the geometry of the microwave apparatus on the rotomolding process.

**Figure 3:**
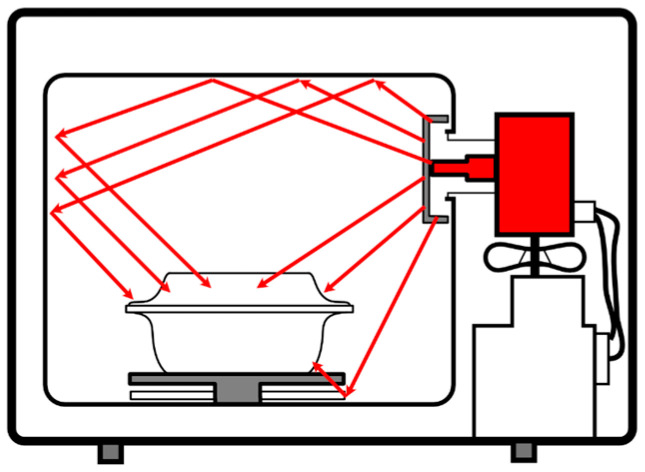
Sketch of MW propagation (as red rays) inside the oven. The direction is indicated by red arrows.

Special care should indeed be employed to find the best focus position in the microwave oven in relation to the geometry of the mold, this to avoid unwanted reflection of the waves as they can cause a failure in the manufacturing process (Figure 3).

Considering the magnetron (i.e., the MW generator) position, we always needed to ensure that the maximum of the reflected radiation could be absorbed by the active material of the mold, as reported in [23,24], while also ensuring the maximum heat exchange in order to ensure the repeatability of the experiments.

**Figure 4:**
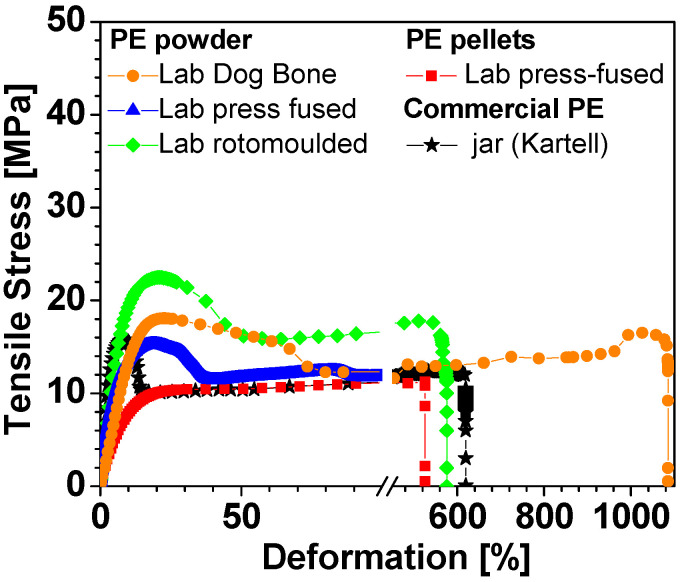
Representative stress–strain curves of several PE dog-bone specimens obtained from different processes: cut from commercial PE jar (Kartell 1000 mL), black star; cut from lab press-fused Plastene powder, blue triangle; cut from lab press-fused Riblene pellets, red square; lab press-fused Plastene powder into dog-bone mold, orange circle; cut from lab MW-rotomolded Plastene powder, green diamond.

The graph in Figure 4 shows the r.t. stress–strain curves obtained by uni-axial tensile measurements corresponding to the most representative samples of the various PE tested. Table 7 summarizes the ensuing tensile data relating to the average values and standard deviation of five specimens for each type of PE investigated.

Looking at Figure 4 and Table 7, a very similar behavior can be observed for all the samples. In particular, for the value of strength as well as for the elongation one, both referred at the break. The elongation at the break is more affected by the presence of defects in the structure [25,26], and then some samples break at ε*_b_* values less than 200% and others resist until 1100%. The lab MW-rotomolded sample (green diamond) shows a very similar behavior to that of the commercial PE of the Kartell jar (black star). Without going into the details of the results obtained with tensile measurements, as it is beyond the scope of this work, it is possible to conclude that the microwave-assisted rotational molding process, although not yet studied and optimized in detail, proves to be competitive with the classic molding techniques.

**Figure 5:**
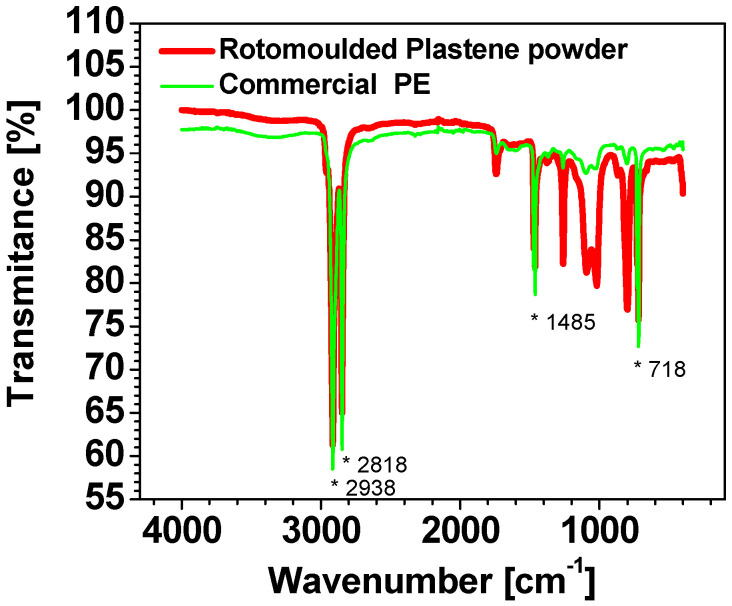
ATR-FTIR spectra of MW-rotomolded (red curve) and commercial (green curve) PE samples. The characteristic PE absorbance bands are located at 2914, 2847, 1470, and 718 cm^−1^ and marked with *.

FTIR techniques, as reported by Almond et al. [27], can be used to spot the degradation of the polymer highlighting the presence of groups derived from oxidation (such as carbonyl and hydroxyl groups). In our case, the MW-assisted rotomolding process did not degrade the PE material, as shown (Figure 5) by the absence of the abovementioned groups in the ATR-FTIR spectrum and by its substantial overlapping to that of the commercial PE sample. The only differences, visible between 900 and 1300 cm^−1^, are ascribed to the peaks of surface-adhered silicone oil used as de-molding agent [28].

**Figure 6:**
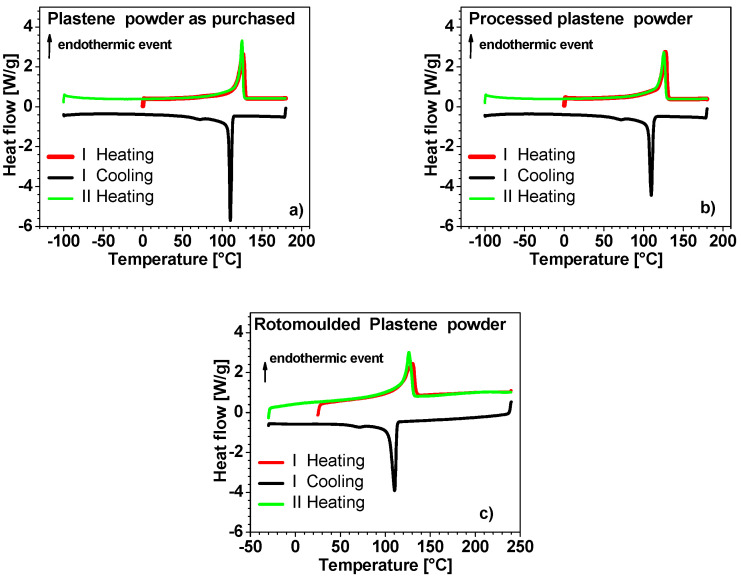
Differential scanning calorimetry graphs relatively to: (**a**) Plastene powder as purchased, (**b**) Plastene after press-fusion and (**c**) MW-rotomolded Plastene.

Figure 6 show the thermograms resulting from DSC analysis. The canonical peak of PE melting in the interval between (100–135 °C during the I and II heating) is recognized for all the samples in the exam. An analogous observation can be performed for the crystallization peak (centered around 110 °C). No other peaks are present. The values of enthalpy of fusion are comparable (around 150 J/g), again suggesting that no degradation occurred during processing. The high crystallinity degree (X_c_ 50–52% taking 293 J g^−1^ as the fusion enthalpy value of a perfect polyethylene crystal) [29,30] of Plastene grade does not permit us to detect the transformation of the amorphous phase from rigid glass and viscous liquid phase (Tg = −80 °C) [30,31].

**Figure 7:**
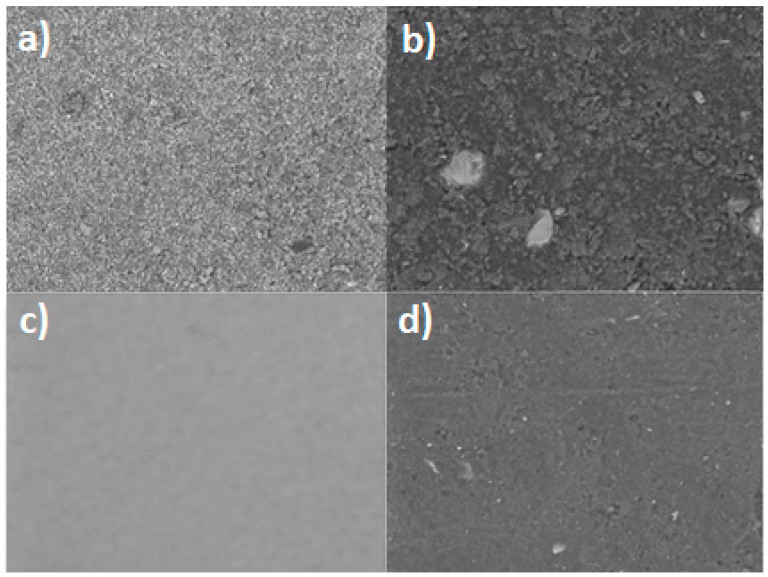
SEM images of the dispersion into the silicone resin of (**a**) Fe_2_O_3_, (**b**) Fe_2_SiO_4_, (**c**) TiO_2_ and (**d**) SiC.

Furthermore, in order to have more information on the different MW-active coating materials used to cover aluminum, stainless steel and glass molds, each coating material was analyzed by SEM-EDX analysis. In Figure 7, the SEM images of the different MW-active coatings are reported: (a) IO (iron oxide), (b) ISF (iron silicate), (c) TO (titanium oxide) and (d) SCF (silicon carbide). Among the four materials, the iron silicate-based one appears to have a more inhomogeneous surface.

**Figure 8:**
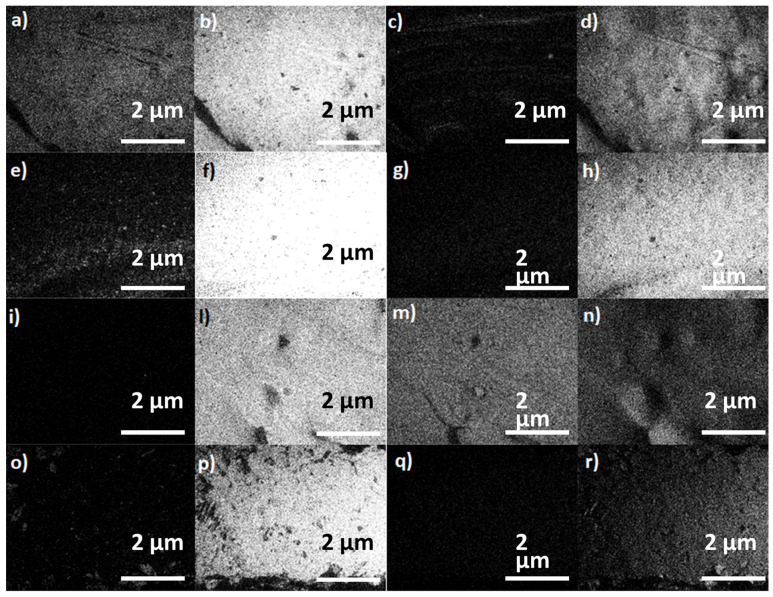
Elemental EDX analysis on the four different MW-active materials dispersed into silicone resin matrix: (**a**) Fe, (**b**) Si, (**c**) Ti, (**d**) O into Fe_2_O_3_, (**e**) Fe, (**f**) Si, (**g**) Ti and (**h**) O into Fe_2_SiO_4_, (**i**) Fe, (**l**) Si, (**m**) Ti and (**n**) O into TiO_2_, (**o**) Fe, (**p**) Si, (**q**) Ti and (**r**) O in to SiC.

In Figure 8, the images corresponding to the EDX analysis of the four samples are reported. These confirm the homogeneous dispersion of the powders and their composition. Only the significative elements are reported for each formulation following this order: Fe_2_O_3_, Fe_2_SiO_4_, TiO_2_ and SiC. The formulations are, respectively,

- Fe in (a), (e), (i) and (o);
- Si in (b), f), (l) and (p);
- Ti in (c), (g), (m) and (q);
- O in (d), (h), (n) and (r).

From EDX images, we can conclude that Si and O are present, as expected, in all the examined composite materials. Ti is obviously present only in (m), while Fe is present in (a) and (e).

Since the melting temperature range of PE is around 130 °C, we checked the chemical stability in air of the MW-active coating materials dispersed into the resin matrix in the range temperature between 0 °C and 300 °C (at the heating and cooling rate of 10 °C/min) by DSC. All the materials did not show any critical issue.

## 4. Conclusions

The focus of this article was the formulation of composite materials responsive to microwave heating as coatings for molds used in the classical rotomolding process to convert it to a microwave-assisted rotomolding technique, thus making the process more efficient.

For this purpose, we used formulates based on a methyl phenyl silicone resin and different inorganic susceptible powders.

The elastomeric material was confirmed to be highly resistant to heat and did not present any degradation during the processes.

The best MW-susceptible inorganic compound used results to be Fe_2_SiO_4_, followed by SiC and Fe_2_O_3_, regardless of the material used for the mold (stainless steel, aluminum and glass were tested).

The chemical nature of the composites prepared ensure that in the presence of damage, the composite material can be easily removed or repaired with subsequent additions of new material.

The measurements of the absorbed power reported showed that dielectric heating saves time and energy if compared to the conventional electric resistance heating process.

The results of the tensile test performed according to ISO 527 (1–5) showed the efficiency of the innovative MW-assisted RM process of PE powder, because the performances are comparable to those shown by the commercial PE jar (Kartell) obtained with a classic molding process.

Finally, it should be considered that the MW-RM process is not limited to PE plastic but can be adapted to any plastic or its composite whose molecules do not have a dielectric moment.

## Figures and Tables

**Table 1:** List of MW-susceptible inorganic compounds (MWSIC) investigated in this work.

MWSIC Formula	Labeled As	Description
SiC	SCF	Silicon carbide 35 μm grain size
SiC	SCC	Silicon carbide 70 μm grain size
Fe_2_SiO_4_	ISF	Iron silicate 35 μm grain size
Fe_2_SiO_4_	ISC	Iron silicate 500 μm grain size
Fe_2_O_3_	IO	Iron oxide powder < 5 μm, ≥99%
TiO_2_	TO	Titanium oxide ≥ 99%
BaTiO_3_	BTO	Barium titanate powder < 3 μm, ≥99%

**Table 2:** Literature dielectric data of the MW-susceptible inorganic compounds investigated as suitable materials for molds coating in MW-assisted RM and of other reference materials.

Material	Measure at	Dielectric Constant *(k*′)	Dielectric Loss *(k*″)	Loss Tangent *(k*″/*k*′)
Fe_2_SiO_4_ [15,16]	25 °C, 10 GHz	5.77	0.01 **	0.0018
SiC [12]	25 °C, 3–10 GHz	10–60	0.01–36 **	0.001–0.58
TiO_2_ [11,12,13,14]	20/25 °C, 4 GHz	80–170	0.008–0.017 **	0.0001
Fe_2_O_3_ [17]	20/25 °C, 3 GHz	6–50	1–4	0.2–0.6
BaTiO_3_ [21]	30 °C, 1 MHz	2200	150	0.068 **
H_2_O [12]	20 °C, 0.1/2.5 GHz	78.1/80.1	3.6	0.016/0.123
SiO_2_ [12,20]	25 °C, 8.5 GHz	3.5–4	0.0008 **	0.0002
Na_2_SiO_3_ [16,17]	25 °C, 8.5 GHz	5.84	0.041 **	0.0070
PE [12,20]	25 °C, 2.5 GHz	2.444	0.002 **	0.0010 (2.6 *)
PVC [12,20]	30 °C, 0.01/2.5 GHz	3/2.666	0.018/0.04	0.001/0.013
PTFE [22]	25 °C, 8.5 GHz	2.058	0.0022 **	0.00108
Silicon RTV 521	23 °C, 8.5 GHz	3.31	0.085 **	0.0257

* Van der Graaff irradiated sample; ** valued by Equation (5).

**Table 3:** Transferred heat calculated with Equation (5).

MW-Active Compound	Q [J] in Cycle at: 750 W, t = 5 min	Q [J] in Cycle at: 750 W, t = 1.5 min
ISC	1000	125
ISF	700	84
SCC	505	63
SCF	500	65
TO	170	75
IO	330	117
BTO	170	84
IO:BTO (1:1)	167	84

**Table 4:** Absorbed power calculations with Equation (6) for the MW irradiation @ 300 W (t < 300 s).

Mold Material (Time)	Absorbed Current [A]	Grid Voltage [V]	Time [s]	Absorbed Power [Wh]
AL (780 s)	5.60	225	286	100
SS (780 s)	5.40	224	286	96
GL (780 s)	5.70	226	286	102

**Table 5:** Absorbed power calculations with Equation (6) for the resistance irradiation (780 and 960 s).

Mold Material (Time)	Absorbed Current [A]	Grid Voltage [V]	Time [s]	Absorbed Power [Wh]
AL (780 s)	4.00	226	780	196
SS (780 s)	4.01	224	780	195
GL (780 s)	4.01	225	960	241
GL (960 s)	4.03	223	780	195

**Table 6:** Weight values of PE products obtained with both MW and Resistive method.

	MW Process	Resistive Process
Material mold	Weight [g]	Weight [g]
SS	14.4 (e)	13.3 (f)
GL	15.0 (c)	8.2 (d)
AL	15.0 (a)	8.2 (b)

**Table 7:** Data from r.t. uni-axial tensile measurements. # denotes the commercial sample is considered as produced by classic molding process, i.e., not using microwave-assisted rotomolding.

Sample/Preparation Process	Tensile Modulus [MPa]	Tensile Strength at Break [Mpa]	Elongation at Break [%]
Riblene (LDPE) [23]	120–550	32–60	450–810
Commercial PE/#	489 ± 115	12.5 ± 1.6	435 ± 178
Press-fused pellets	116 ± 12	11.6 ± 1.3	520 ± 217
Press-fused powder	291 ± 23	8.5 ± 2.6	160 ± 80
Lab Dog Bone	190 ± 35	16. ± 3.4	1100 ± 491
Lab MW-rotomolded	287 ± 78	21.7 ± 3.2	320 ± 201

## Data Availability

Data sharing not applicable.
